# Structure–Property Relationships Governing Rheological, Damping, and Thermal Behaviour of Immiscible Natural Rubber/Nitrile Rubber Blend Nanocomposites

**DOI:** 10.3390/polym18040427

**Published:** 2026-02-07

**Authors:** Martin George Thomas, Sanitha Vasudevan, Hanna J. Maria, Ange Nzihou, Roberto Scaffaro, Marco Morreale, Sabu Thomas

**Affiliations:** 1 Department of Physics and Electronics, Christ University, Bangalore 560029, India; martin.george@christuniversity.in; 2 International and Inter University Centre for Nanoscience and Nanotechnology (IIUCNN), Mahatma Gandhi University, Kottayam 686560, India; sanithathadaqt@gmail.com (S.V.); hannavidhu@gmail.com (H.J.M.); 3 Trivandrum Engineering, Science and Technology (TrEST) Research Park, Thiruvananthapuram 695016, India; 4 IMT Mines Albi, RAPSODEE CNRS UMR 5302, Université de Toulouse, Campus Jarlard, F-81013 Albi Cedex 09, France; ange.nzihou@mines-albi.fr; 5 Department of Engineering, University of Palermo, Viale delle Scienze, 90128 Palermo, Italy; roberto.scaffaro@unipa.it; 6 Department of Engineering and Architecture, Kore University of Enna, Cittadella Universitaria, 94100 Enna, Italy; 7 School of Energy Materials, Mahatma Gandhi University, Kottayam 686560, India

**Keywords:** nitrile butadiene rubber, natural rubber, elastomer blends, nanocomposites, viscoelastic properties, thermal properties

## Abstract

Polymer nanocomposites have been attracting significant interest over the last three decades. One of the most intriguing applications is related to the preparation of clay-filled nanocomposites based on rubber blend matrices. Although several studies already exist on the subject, there is limited information available regarding their rheological, thermal, and, particularly, damping behaviour of rubber blend systems. In this work, the rheological, viscoelastic, and thermal behaviour of a natural rubber/nitrile rubber (NR/NBR) blend nanocomposite containing organically modified nanoclay was systematically investigated, and the damping characteristics were also assessed. At a lower nanoclay concentration (5 phr), network formation through filler–filler and filler–polymer interactions led to partial immobilization of polymer chains, resulting in a pronounced increase in viscosity and enhanced viscoelastic response. In contrast, at higher nanoclay loading (10 phr), strong agglomeration of filler particles occurred, corresponding to a stacked clay morphology, which hindered effective filler–filler network formation and weakened filler–polymer interactions, leading to lower viscosity and reduced damping efficiency. The blend composition and filler content were found to significantly influence the investigated properties, especially the hysteresis loss and the thermal conductivity, which is explained by matrix–filler interactions and the resulting morphology of the system.

## 1. Introduction

Polymer composites and blends provide effective strategies for engineering materials with tailored properties [1,2,3].

Furthermore, nanocomposites based on elastomeric matrices have gathered significant attention due to their ability to achieve superior mechanical, rheological, viscoelastic, and thermal properties compared to traditional composites [4,5,6,7].

Elastomer nanocomposites can exhibit significant property enhancements when filler particles are introduced, but understanding these improvements requires detailed insights into the polymer/filler network.

Rheological and viscoelastic properties of the investigated system are critical, as they define material behaviour under shear forces, with several implications across diverse industrial applications, fundamental in enhancing quality and optimizing the actual processing conditions. Filler reinforcement mechanisms can be analyzed through strain sweep studies, which reveal a strain-dependent variation in storage and loss moduli, known as the Payne effect, a distinctive characteristic of filled rubbers at low strain amplitudes [8,9], which is of significant current interest, as recent studies highlight [10]. Furthermore, stress softening, or hysteresis loss, during cyclic loading and unloading is another critical feature, highlighting energy dissipation, which is important in several industrial applications.

The thermal behaviour of polymer blends is equally fundamental for material design. Key factors influencing rubber product longevity include matrix, additives, network structure, and thermal history. Thermal conductivity is particularly important, as heat buildup from repeated deformation or friction can reduce material efficiency and lifespan. The addition of suitable filler additives could enhance thermal conductivity, providing efficient heat dissipation pathways and extending product durability [11,12]. Thermophysical properties like thermal conductivity, diffusivity, and specific heat determine heat transfer performance and guide material optimization. Filler dispersion further influences thermal properties, and measurement of thermal conductivity can provide semi-quantitative estimates of dispersion degree [13,14]. While thermal conductivity studies can be useful regarding the processing, determination of the glass transition temperature (Tg) is important in order to assess the miscibility of the polymer blends. Immiscibility is marked by distinct Tg values for each polymer, while partial miscibility leads to Tg shifts, narrowing the interval between transitions. Fully miscible blends exhibit a single Tg.

Research on thermal properties of polymer blends has shown various results [15,16,17]. For instance, Saxena et al. [18] observed enhanced thermal conductivity in styrene-butadiene rubber (SBR) with nitrile rubber (NR) latex waste fillers. Li et al. [19] noted a gradual thermal conductivity increase in nano-zinc oxide/emulsion polymerized butadiene-styrene rubber (ESBR) composites, aligning with theoretical predictions. Zhou et al. [20] found that carbon nanotube (CNT) addition improved thermal conductivity in spray-dried CNT/SBR composites.

Studies on carbon black [21] and alumina-based fillers [22] have highlighted their role in heat dissipation, with carbon black improving the thermal conductivity and mechanical properties [23], and nano-alumina outperforming micro-alumina.

Thermal degradation and dynamic mechanical properties of rubber blends were investigated by several research teams [24,25,26]. Thermal stability improvements in polymer blends have been linked to enhanced morphological stability and interfacial interactions. For example, Jana and Cho [27] attributed higher stability in polyurethane nanocomposites to covalent bonding with functionalized multiwalled carbon nanotubes, improving dispersion and resistance to decomposition. In general, nanoparticles might improve the thermal stability [28]. Balachandran et al. [29] studied the effect of expanded graphite (EG) on ethylene-propylene-diene (EPDM)/fluorocarbon elastomer (FKM) blends, compatibilized (via maleic anhydride grafted EPDM (MA-g-EPDM) rubber) or uncompatibilized, investigating the thermal stability via thermogravimetric analysis (TGA).

Sivaramakrishnan et al. [30] studied the effect of modified nanosilica (mNS) on natural rubber (NR) and acrylonitrile butadiene rubber (NBR) composites, with epoxidized natural rubber (ENR) as compatibilizer. They found that mNS content up to 10 phr improved the mechanical resistance, especially in the case of 70/30 NR/NBR blends with 6 phr of mNS, and this was attributed to the high surface area and uniform distribution of mNS.

Organomodified nanoclays, characterized by their high aspect ratio and large surface area, can facilitate strong interfacial interactions with the polymer matrix [31,32]. These interactions can lead to improved dispersion within the elastomer blend, directly influencing the composite rheologic and thermal properties. In this context, blends of natural rubber (NR) and nitrile butadiene rubber (NBR) could offer a versatile platform for designing advanced materials, combining the excellent elasticity of NR with the chemical resistance and thermal stability of NBR [33,34,35,36]. Furthermore, considering that a NR/NBR matrix could achieve a suitable compatibility with organomodified nanoclay, significant improvements in thermal conductivity and stability could be obtained.

However, it can be noticed from the above analysis of the Literature that, to our best knowledge, there is not much information available regarding the rheological, thermal, and damping behaviour of NR/NBR blends; in particular, there is a lack of comprehensive studies on the rheological, thermal, and, especially, damping properties of NR/NBR/organoclay systems.

In this study, therefore, we aim at addressing this gap by investigating NR/NBR blend nanocomposites containing organo modified nanoclay, and focusing on the rheological and viscoelastic behaviour, with special attention regarding the damping properties and thermal conductivity. The effect of filler loading, blend composition, and clay modification was analyzed, with the objective to advance understanding of nanoclay reinforcement and optimize blend compositions for further industrial applications, especially those where suitable damping properties are required but face with the limitations of common damping materials such as unsuitable weight and poor thermal properties. This study, moreover, clarifies how filler loading and blend composition govern the rheological, viscoelastic, and damping behaviour of NR/NBR nanocomposites, enabling optimized material design for improved performance.

## 2. Materials and Methods

### 2.1. Materials

The NR used in this work was commercially produced from the latex of Hevea Brasiliensis by Rubber Board (Kottayam, Kerala, India) and its main characteristics are reported in Table 1.

The NBR, an oil-resistant synthetic rubber produced from a copolymer of acrylonitrile and butadiene, was supplied by Eliokem Industries Ltd. (Mumbai, India). Its main characteristics are reported in Table 2.

The crosslinking agent used was sulphur (S), supplied by Merck India Ltd. (Mumbai, India). As the activator, ZnO, supplied by Lanxess Pvt Ltd. (Cologne, Germany), was used. The primary and secondary accelerators viz l-2-cyclohexyl benzothiazyl sulphenamide (CBS) and tetra methyl thiurium disulphide (TMTD) were purchased locally.

The nanocomposites were prepared using organically modified montmorillonite clays as nanofillers.

In more detail, Cloisite 10A (provided by Southern Clay Products, Mumbai, India) was used. Cloisite 10A is an organically modified montmorillonite with dimethyl, benzyl, and hydrogenated Tallow alkyl tail modification, with cation exchange capacity (CEC) equal to 125 meq/100 g and an average dry particle size in the range of 2 µm–13 µm. Main properties are reported in Table 3.

For comparison purposes, an organically modified kaolin nanoclay with mercapto silane modification, having 60–80 nm average particle thickness, 200 nm average diameter, and specific surface area of 3.06 m^2^/g, was also used, and labelled here as O_2_K. The average composition was 45% SiO_2_, Al_2_O_3_ 38%, Fe 0.5%, TiO_2_ 25.5%, CaO 0.6%, MgO 0.07%, Na_2_O-0.1%, and K_2_O-0.03%. The silane content for modification was 0.5%.

### 2.2. Processing

Before compounding, the nanoclays were dried in a vacuum oven at 80 °C for about 16 h. Compounding was then carried out in a laboratory two-roll mill, with initial temperature of 80 °C and the formulations given in Table 4.

The samples obtained were compression-moulded at 150 °C with a t_90_ curing time obtained from tests in an oscillating disc cure metre according to ASTM D2084 [37].

Solution mixing involved three major steps: dispersion of nanofillers in toluene by mechanical mixing, magnetic agitation or sonication, and mixing of dispersed nanoparticles into the polymer matrix at room or elevated temperatures. The dispersed nanofiller was mixed with 10 g of NR and 10 g of NBR solutions, previously mixed using mechanical stirring. The mixture was then further mechanically stirred at 5000 rpm for a duration of 2 h at room temperature. The nanocomposite was finally obtained by casting the mixture. This was then further mixed with the remaining part of the rubber, and the compounding was carried out as in the case of dry mixing, as described in Table 4.

In summary, several systems were prepared, where the NR/NBR blend composition might range between 30 and 70%, while clay amount might be present as 1, 2, 5 or 10 phr.

**Scheme 1:**
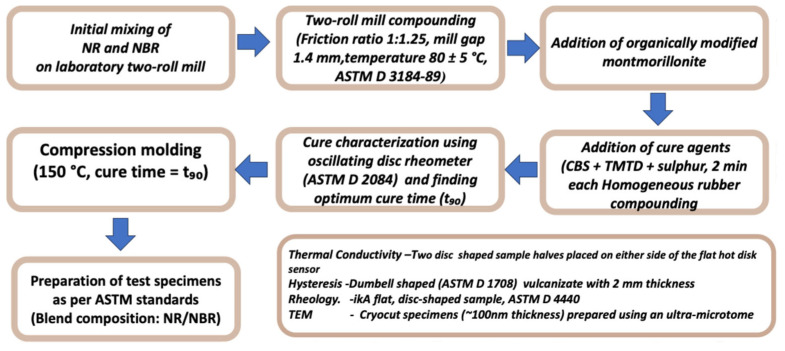
Flow chart of the preparing the blend nanocomposite [37,38,39,40].

The entire process is shown in Scheme 1.

The rheological measurements were performed using a stress-controlled rheometer (REOLOGICA Instruments AB, Lund, Sweden). Frequency sweep measurements were carried out over a frequency range of 0.01 Hz–40 Hz. The cure characteristics of the blend nanocomposites were studied at 100 °C under oscillation strain control.

Stress–strain and hysteresis tests were performed using a Tinius Olsen H50KT Universal Testing Machine (Tinius Olsen Inc., Horsham, PA, USA, supplied by AIMIL Ltd., New Delhi, India) controlled by Test Navigator software. Dumbbell-shaped (ASTM D1708) [39] NR/NBR blend nanocomposite vulcanizates with 2 mm thickness were used. Samples were stretched and released with a 500 mm/min crosshead speed. The three-cycle hysteresis behaviours of blend nanocomposites were investigated by stretching them to 200 or 300% elongation and immediately releasing (unloading). Consecutive hysteresis cycles were initiated after the crosshead returned to the original starting position.

**Scheme 2:**
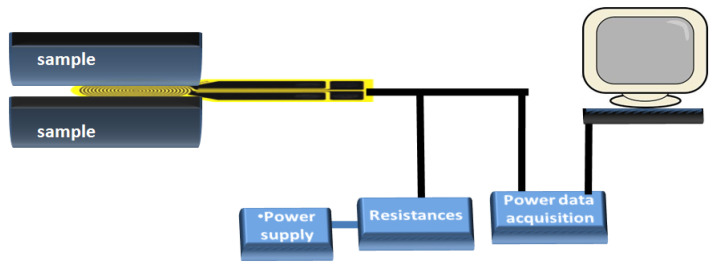
Thermal conductivity experimental set up.

Thermal conductivity was measured through the hot disc method. A probe formed by a double spiral nickel on an insulating support in Kapton or mica (according to the measurement temperature) is positioned between 2 samples of the studied material. Scheme 2 shows the experimental set up. The hot disc measures the conductivity. It is a Transient Plane Source method (TPS), which is suitable to measure thermal conductivity and thermal diffusivity in both insulating and diffusive materials.

The equipment was provided with an electric resistance measurement (Source metre 2400 Keithley), a natural convection airflow oven (Heraeus model T6060, Albi, France), and a data programme for thermal diffusivity (a, m^2^ s^−1^) and thermal conductivity estimation (k, W m^−1^ K^−1^), which allows for the calculation of the volumetric heat capacity (C_P_, J m^−3^ K^−1^). The thermal properties were determined at 25 °C equilibrating time; to ensure the desired temperature, at least three measurements were performed every 30 min.

To assess the quality of filler dispersion and morphological details, the NR/NBR nanocomposites were investigated by means of Transmission electron microscopy (TEM), using a JEOL (JEOL India Pvt. Ltd. Jasola, New Delhi, India) JEM-2100 HRTEM. The micrographs were obtained in point-to-point resolution 0.194 nm, operating at an accelerating voltage of 200 kV. Ultra-thin sections of bulk Cryocut specimens (~100 nm thickness) prepared using an ultra-microtome (Leica, Ultracut UCT, Wetzlar, Germany) were placed on a 300 mesh Cu grids (35 mm diameter) and were analyzed without staining.

## 3. Results and Discussion

### 3.1. Rheological Behaviour

**Figure 1:**
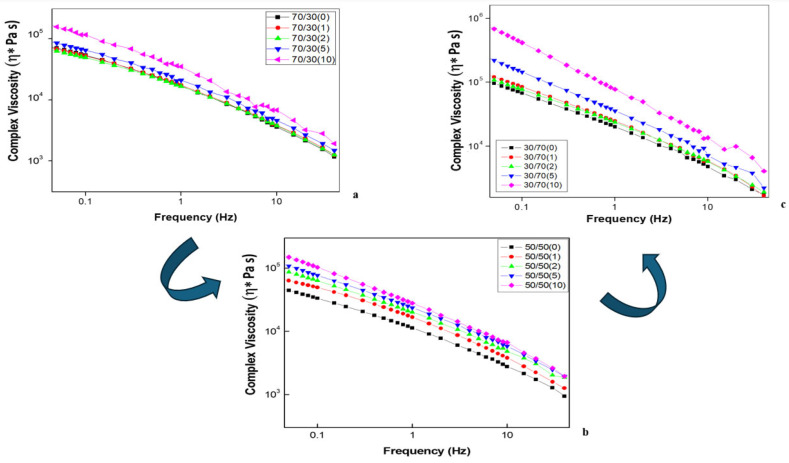
Frequency dependence curves of complex viscosity η* for 70/30 (**a**), 50/50 (**b**), and 30/70 (**c**) NR/NBR blends.

The complex viscosities, η*, of the prepared NR/NBR 70/30 blend nanocomposites (up to 10 phr nanoclay content) are shown in Figure 1a–c.

First, it can be observed that the complex viscosity increases on increasing the nanoclay content, except for lower nanoclay amounts, in agreement with results obtained on similar systems containing nanosized particles [7,41,42]. In all cases, the investigated systems show the typical shear thinning trend, partly except for 10 phr nanocomposites, where stronger matrix–filler interactions seem to occur even at higher frequencies, those typical of most industrial processing operations.

**Figure 2:**
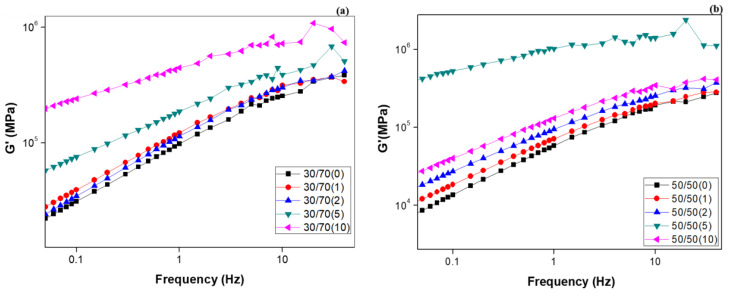
Storage modulus vs. dynamic amplitude curves for 30/70 (**a**) and 50/50 (**b**) NR/NBR blends with different filler loadings.

**Figure 3:**
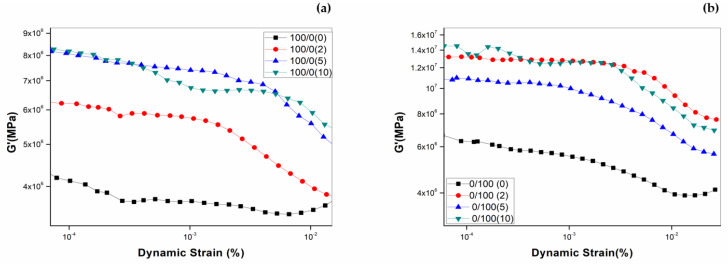
Storage modulus vs. dynamic strain curves for 100/0 (**a**) and 0/100 (**b**) NR/NBR blends with different filler loadings.

In order to assess the influence of blend composition on the rheological properties of the obtained nanocomposites, 50/50 and 30/70 NR/NBR blends were subjected to the same rheological measurements, and the results are shown in the following Figure 2 and Figure 3, respectively. Shifting from 70/30 to 50/50 composition does not significantly change the overall behaviour, except for the differences being harsher on increasing the nanoclay content, and especially all of the nanocomposites showing higher viscosities than the unfilled blend. This suggests stronger matrix–filler interactions at the molten state in all the nanocomposite systems, if compared to the 70/30 systems. This seems to be confirmed by the slightly higher slopes at low frequencies.

The trend seems, on average, confirmed also upon increasing the NBR amount to 70%, with significant increase in the overall viscosities for all of the investigated systems, and slightly increased non-Newtonian behaviour.

Figure 2a,b show the storage modulus, G’, as a function of the frequency for the 50/50 and 30/70 systems, which were those that suggested, from the viscosity curves, higher degrees of matrix–filler interaction. In fact, the significant increase in the storage modulus with the nanoclay content suggests an increased solid-like behaviour through the formation of physical connectivity or a percolated network between clay layers [43]; this is further confirmed by the reduced slope at lower frequencies which indicates an increased elastic behaviour on increasing the nanoclay content [44,45].

### 3.2. Effect of Nanoclay on the Polymer–Filler Network Formation

In elastomer nanocomposites, the linear viscoelastic region can be determined by observing the trend of dynamic viscoelastic properties (such as the storage modulus) against stress strain, in strain sweep tests.

Actually, at higher strains, rigid filler layers tend to be destroyed and the same will consequently happen to the filler platelet surfaces–polymer chains interactions, leading to non-linear strain dependence of the storage modulus [46]. This is known as “Payne Effect” [8]. Of course, this effect will also depend on several factors related to the polymer–filler interaction, such as the interaction energy, chain entanglements, and the actual contact surfaces [10,47,48]. The effect will we more noticeable in the presence of stronger inter-particle forces, as in the case of small distances between filler particles and presence of a strong filler–filler network [49,50], as reported by Poikelispää et al. [50] on NR/NBR blends filled with either carbon black or carbon nanotubes, where Payne effect was stronger for the former, while the latter showed better rubber–filler compatibility.

In the case of the systems investigated in the present work, Figure 3 shows the trend of G’ vs. strain for 100/0 and 0/100 NR/NBR blends at different filler loadings. As expected, the unfilled systems show practically no Payne effect, i.e., almost steady G’ upon changing the applied strain. Completely different behaviour is shown by the filled systems, with clear non-steady trends at higher strains, attributable to complex effects including filler–filler interaction, polymer network interactions, and hydrodynamic effects in rubber structure.

For pure elastomers nanocomposites, it can also be observed that (Figure 3a,b) the Payne effect is prominent, or the amplitude of Payne effect increases with filler loading. This can be due to the good dispersion of nanoclay in the polymer matrix.

Attempts were made to evaluate the Payne effect for the NR/NBR blend nanocomposites; however, the obtained G′–strain data were not sufficiently reproducible to allow a meaningful interpretation. This may be due to the complex co-continuous morphology of the blends and competing filler–polymer and filler–filler interactions. Therefore, Payne-effect analysis is presented only for the neat rubber nanocomposites, while the behaviour of blend nanocomposites is discussed based on storage modulus and hysteresis results.

**Figure 4:**
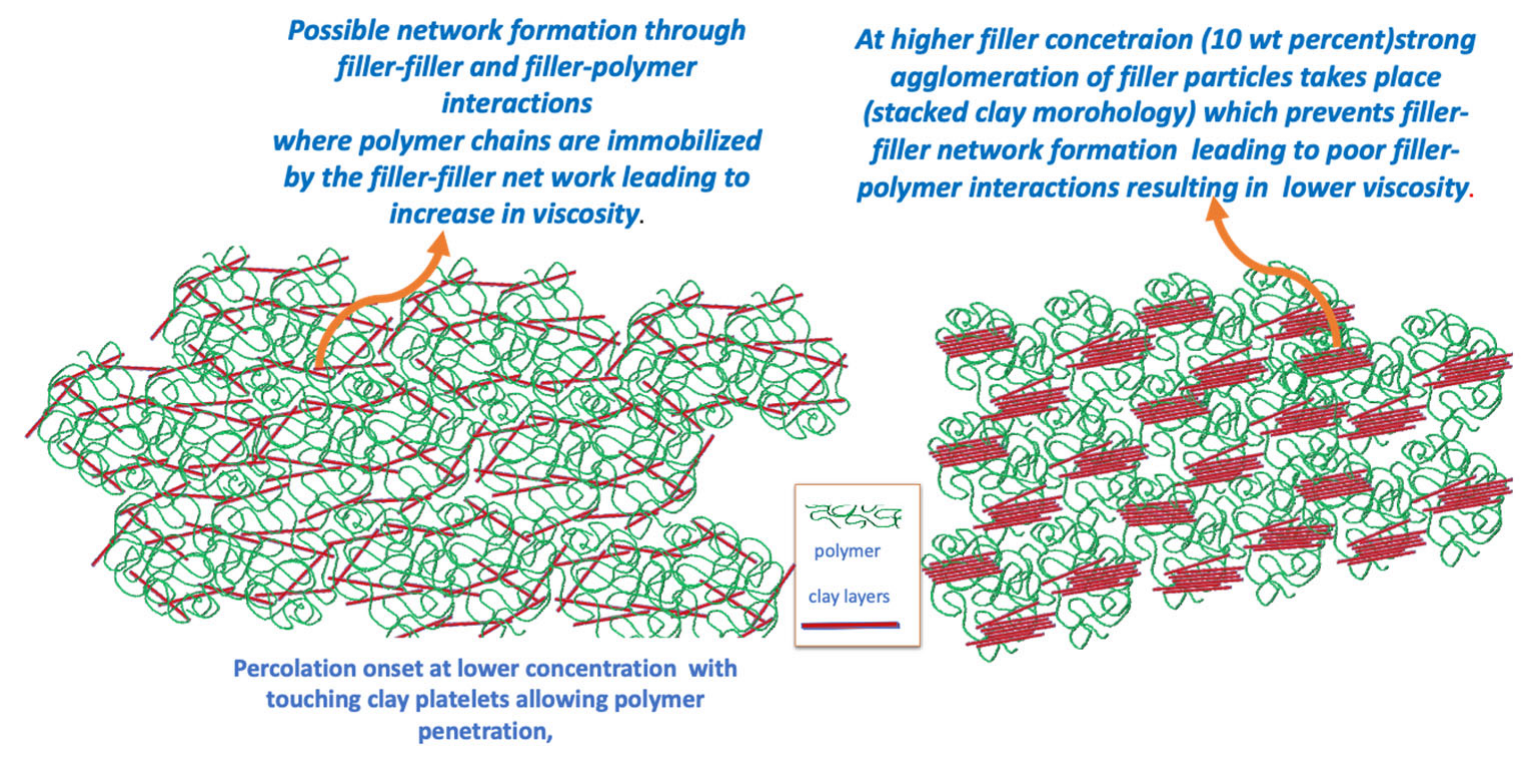
Schematic representation of filler network formation at moderate loading and agglomeration at higher loading.

In fact, the rheological percolation generated by the clay is the sudden transition of an elastomeric composite from a liquid-like to a solid-like behaviour as the clay concentration reaches a critical concentration. This process is caused by the formation of a three-dimensional, space-filling clay–clay network of interconnected clay particles within the polymer matrix, as shown in Figure 4. However, at 10 phr filler loading, the system tends to develop very strong filler–filler agglomeration, as shown in the same Figure 4, especially in a co-continuous NR/NBR blend. This excessive filler content disrupts the uniform filler network and reduces effective polymer–filler contact. As a result, the effective reinforcement decreases, leading to a comparatively lower storage modulus despite the higher filler content. Such non-linear reinforcement behaviour is well known in filled elastomer systems and is commonly attributed to the transition from an optimal percolated filler network (at lower loading) to agglomeration-dominated structures (at higher loading).

The above proposed explanation accounts also for some apparently unexpected trends observed in the complex viscosity and storage modulus curves shown previously.

### 3.3. Hysteresis—Damping Efficiency of Blend Nanocomposites

One of the most interesting applications of highly viscoelastic materials is related to damping devices.

Common damping materials, however, tend to show several shortcomings, such as unsuitable weight, unsatisfactory long-term reliability, and poor thermal properties (such as low thermal conductivity and unsatisfactory performance at extreme temperatures).

Therefore, in attempt to enhance the properties of “base” damping materials, research has focused on possible modifications such as blends, composites, and/or (as in the case of the present work) nanocomposites. In any case, keeping and/or achieving good values of fundamental properties related to damping efficiency, such as the modulus and hysteresis cycle, is crucial. Actually, several fundamental properties like wear, modulus, tear, heat generation, etc. are often correlated with hysteresis loss. Elastomer nanocomposites may show improved hysteresis (and therefore, damping properties) thanks to polymer/filler interfacial interaction and/or slippage and, therefore, energy dissipation [51,52]. It is, in fact, known that an improved polymer/filler adhesion means an improved load transfer and in general, higher strength and stiffness [53,54,55,56,57,58]; on the other hand, interfacial slip resulting in a reduction in the latter properties, would be preferrable as far as higher mechanical damping is sought for. This could be the case of nanoclay, providing a large interfacial contact area, facilitating significant frictional energy dissipation during cyclic loading and unloading.

**Figure 5:**
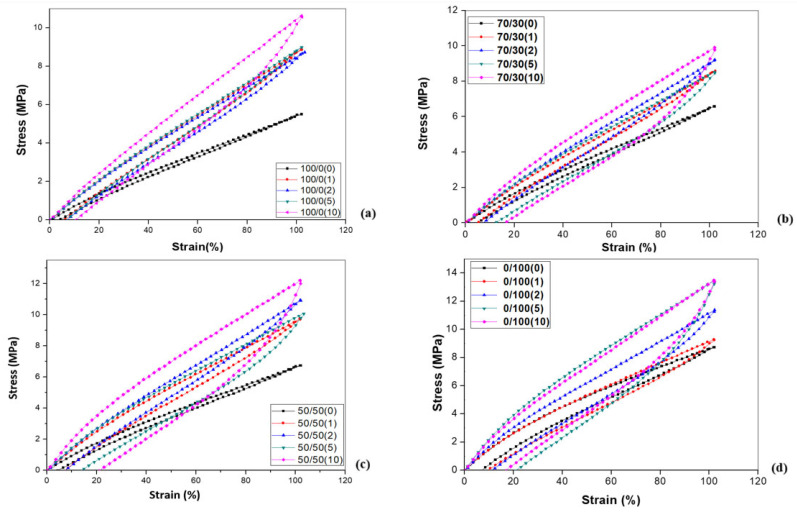
Effect of nanoclay content on single loading–unloading cycles on 100/0 (**a**), 70/30 (**b**), 50/50 (**c**), and 0/100 (**d**) NR/NBR nanocomposites at a constant strain of 100%.

Therefore, single-cycle experiments (Figure 5a,d) were carried out, revealing variations in hysteresis across different compositions and nanoclay loadings. The hysteresis loop area, indicative of damping, correlates with the viscous properties of the vulcanizates and is summarized in Table 5. Generally, hysteresis increases with filler loading, though certain blends deviate due to factors requiring further investigation.

Blend nanocomposites show higher damping than pure nanocomposites, suggesting weaker filler–polymer interactions in the blends compared to organoclay-filled pure elastomers. Furthermore, it is worth noting that hysteresis increases as nanoclay content rises. This can be attributed to energy dissipation mechanisms, including interfacial sliding at filler–polymer interfaces and stick-slip behaviour at filler–filler interfaces. Previous studies [59] also underscore the important role of filler–filler interactions in rubber hysteresis behaviour, which may explain the greater hysteresis observed at higher filler loadings.

### 3.4. Effect of Strain Level and Nanofiller Loading

**Figure 6:**
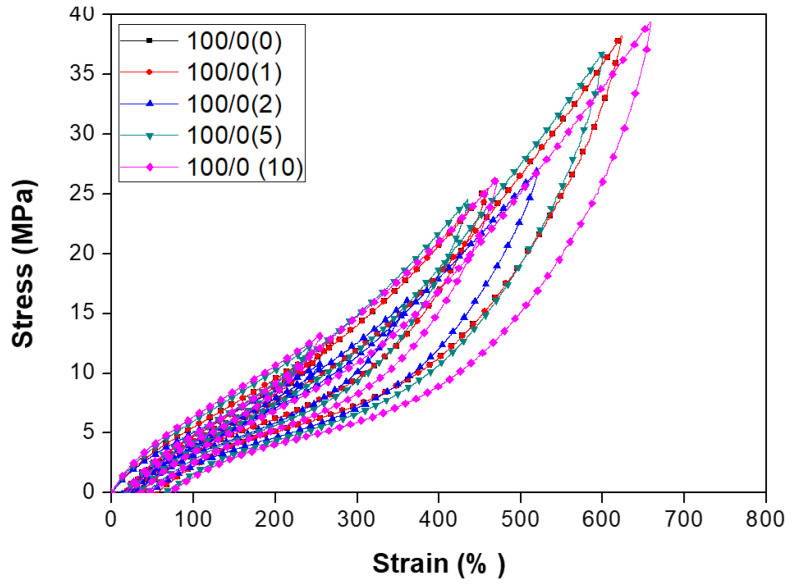
Effect of nanoclay content on three consecutive loading–unloading cycles on 100/0 NR/NBR nanocomposites.

**Figure 7:**
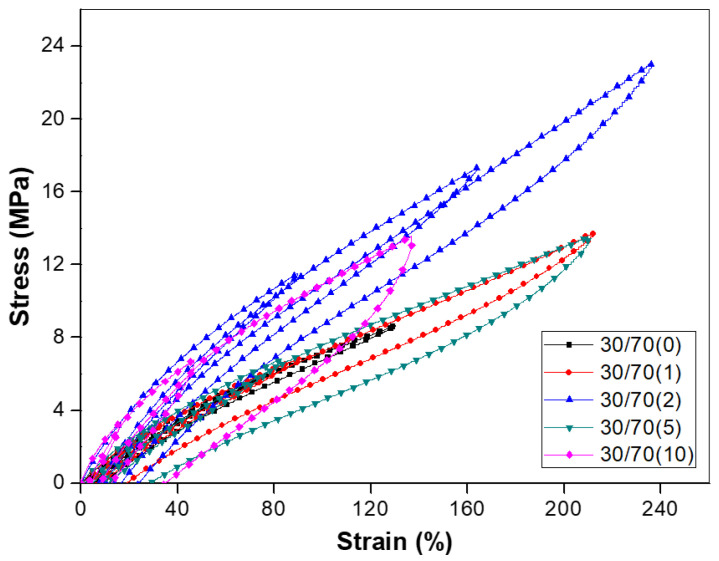
Effect of nanoclay content on three consecutive loading–unloading cycles on 30/70 NR/NBR nanocomposites.

**Figure 8:**
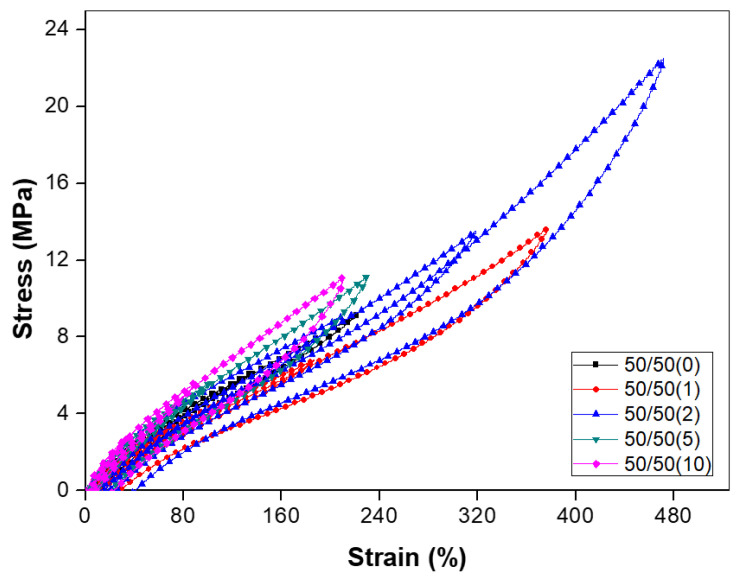
Effect of nanoclay content on three consecutive loading–unloading cycles on 50/50 NR/NBR nanocomposites.

In order to investigate hysteresis loss across various strain levels, stress–strain curve determination was carried out over three cycles. Representative hysteresis loss curves for NR/NBR blend nanocomposites with different filler loadings are thus shown, for the sake of visibility, in Figure 6, Figure 7 and Figure 8. The results demonstrate the expected increase in hysteresis loss on higher cycle number and strain level; however, the rate of increase is not uniform across the strain range.

Most blends exhibit increased hysteresis loss in the second and third cycles. This could be attributed to strain-induced debundling of agglomerates, alongside orientational effects in the stress–strain behaviour. Notably, the magnitude of hysteresis increase is higher for samples with greater filler content, likely due to higher filler–polymer slippage occurring at higher strains during subsequent cycles. Interestingly, varying filler loading does not significantly affect hysteresis in pure NR nanocomposites. For all the nanocomposite systems, damping behaviour is consistent across cycles, except in specific ratios (0/100, 30/70, and 50/50), where a decrease in damping is observed in the third cycle with 2 phr nanoclay loading.

In addition to the single-cycle hysteresis analysis, the effect of repeated loading–unloading was examined for a representative blend nanocomposite. For this purpose, the 70/30 NR/NBR system was selected, and the hysteresis loop areas were evaluated over three consecutive cycles, as reported in the following Table 6. This composition was chosen as it exhibits a typical co-continuous morphology and intermediate damping behaviour among the investigated blends. The results indicate a progressive change in hysteresis with increasing cycle number, reflecting the rearrangement of the filler network and enhanced interfacial slippage under repeated deformation.

### 3.5. Effect of Strain Level and Blend Composition

**Figure 9:**
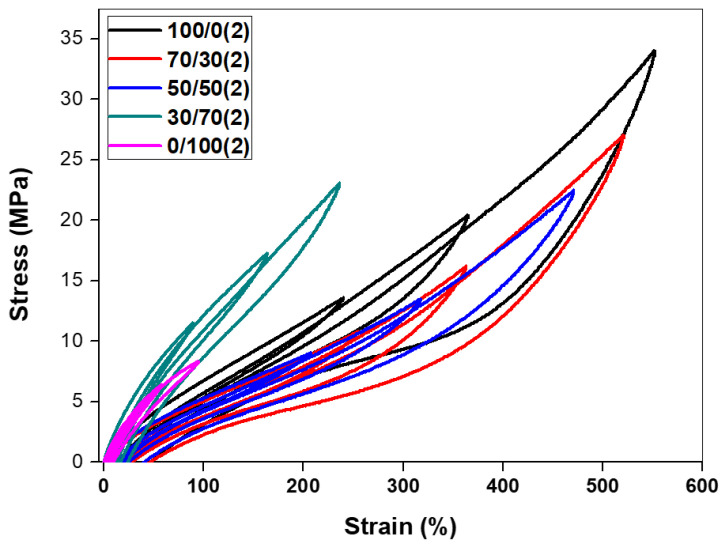
Effect of nanoclay content on three consecutive loading–unloading cycles of different NR/NBR nanocomposites with 2 phr nanoclay.

To assess the effect of blend composition, hysteresis loss at different strain levels was plotted for composites with a specific filler content, i.e., 2 phr nanoclay. The hysteresis curves of the nanocomposites at different blend compositions are shown in Figure 9. The cycle area and, therefore, the dissipated energy was significantly different among the investigated systems and increased on increasing the NR fraction.

### 3.6. Effect of Strain Level and Clay Modification

**Figure 10:**
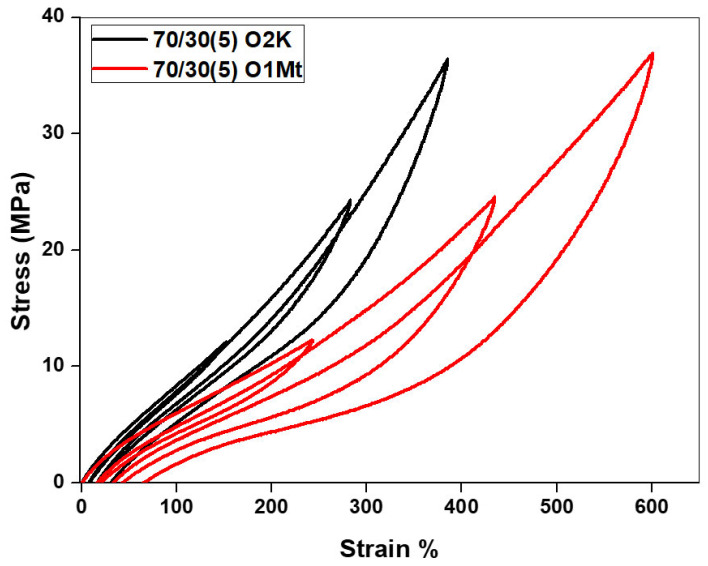
Effect of different types of nanoclay on three consecutive loading–unloading cycles of different NR/NBR nanocomposites with 5 phr nanoclay.

To evaluate the possible influence of different nanoclay modifications, i.e., the Cloisite hereafter labelled as O1Mt, on the hysteresis loss, Figure 10 shows the hysteresis cycles of a 70/30 NR/NBR nanocomposite with the two different nanoclays. It can be observed from the figure that for the blend nanocomposite with mercapto silane modification, the area of the hysteresis loop is smaller. This could be due to the stronger interaction between O2K and NR and the enhanced cross-linking caused by the mercapto silane group. The area inside the loop is given in Table 6 (anticipated in Section 3.4) for further clarification.

### 3.7. Thermal Conductivity: Effect of Blend Composition

In many applications, reducing static charge accumulation on polymer surfaces requires improving thermal conductivity. Elastomers, typically characterized by poor thermal and electrical conductivity, are prone to heat accumulation, a major factor in their ageing under dynamic loading conditions (e.g., in tyres, conveyor belts, and rubber rollers). Enhancing thermal conductivity mitigates this issue by facilitating heat conduction, which is primarily influenced by lattice vibrations, hysteresis loss, and internal polymer friction.

**Figure 11:**
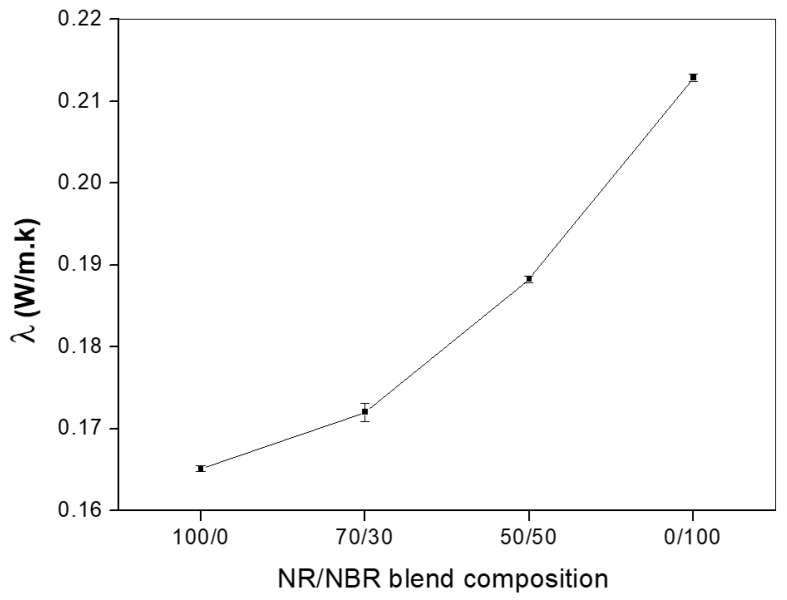
Thermal conductivity vs. blend composition of NR/NBR blends.

A practical approach to improving thermal conductivity is blending elastomers, combining materials with higher conductivity to suit specific applications (e.g., adding an elastomer with higher conductivity). In the NR/NBR blend investigated, the addition of NBR significantly enhances thermal conductivity, as evidenced by an increase proportional to NBR content (Figure 11). This enhancement arises from the intrinsic thermal conductivity differences between the two elastomers: NBR (0.24 W/m·K) and NR (0.148 W/m·K) [60]. The higher polarity of NBR also contributes to its superior conductivity [61].

### 3.8. Thermal Conductivity: Effect of Nanoclay Loading

**Figure 12:**
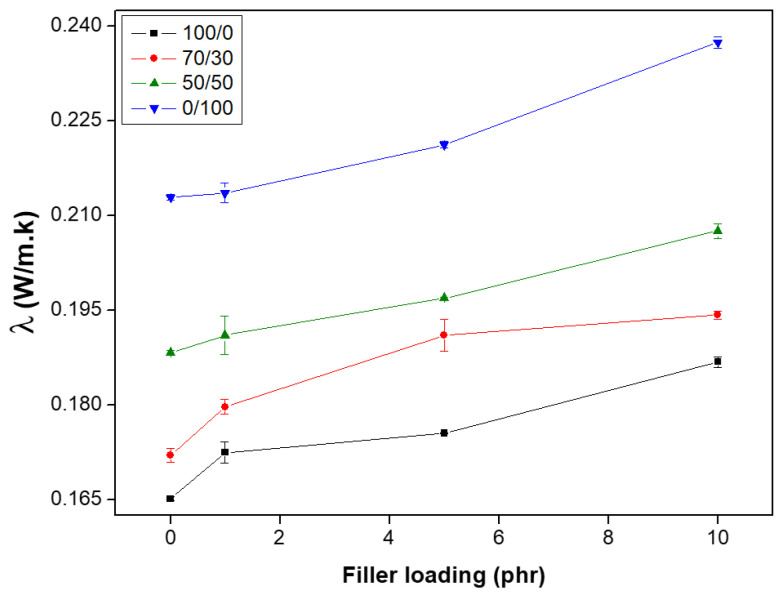
Thermal conductivity vs. filler loading for different systems.

The actual thermal conductivity of a composite depends, of course, on the thermal conductivities of the constituent elements [62]. According to the Agari model [63,64,65], the thermal conductivity of the composite is linearly dependent on the percent volume fraction of the filler. This was assessed by plotting (Figure 12) the experimental values of thermal conductivity vs. filler loading at different NR/NBR blend compositions. It can be observed that a fair agreement with the expected linear dependence occurs, expected on the basis of the higher thermal conductivity of the nanoclay. Furthermore, as previously discussed, due to the higher thermal conductivity of NBR in comparison to NR, the overall conductivity trends are higher on increasing the NBR content.

To achieve high thermal conductivity in composites with low filler content, the filler phase should form a continuous network or create conductive pathways in a co-continuous blend [66]. It is well known that polar systems typically exhibit higher thermal conductivity. In NR/NBR composites with different O1Mt content, the presence of a continuous NBR phase facilitates the formation of conductive chains, explaining the enhanced thermal conductivity in 50/50 blend nanocomposites. Furthermore, on the basis of the previously discussed rheological properties, it is likely that the nanocomposites achieved an intercalated (and maybe exfoliated) morphology, which further improved the heat transfer.

As filler loading increases, O1Mt clay layers establish more contact points, forming conductive networks that significantly boost thermal conductivity. The high surface area of nanoclay (achieved by intercalation and possible exfoliation, as stated previously) further enhances this effect by increasing interfacial contact between the polymer and filler, thereby improving heat transfer [67]. However, at higher filler loadings, agglomeration can occur, which may reduce heat transfer. Notably, particles with an aspect ratio > 1 demonstrate superior directional heat conduction compared to spherical particles (aspect ratio = 1) at equivalent volume fractions [68,69]. This is further confirmed with the morphological images discussed in the following.

### 3.9. Morphological Analysis

**Figure 13:**
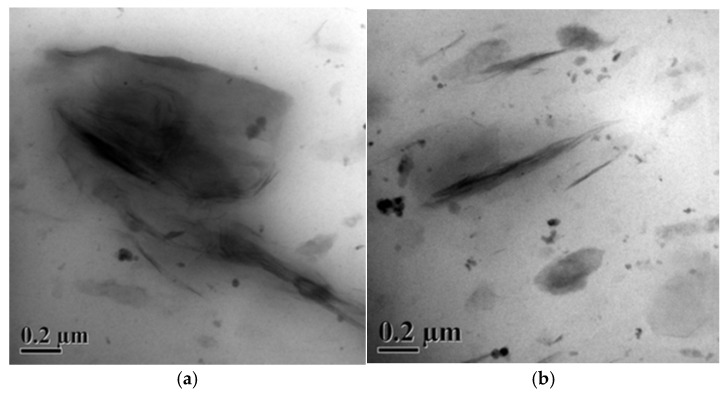

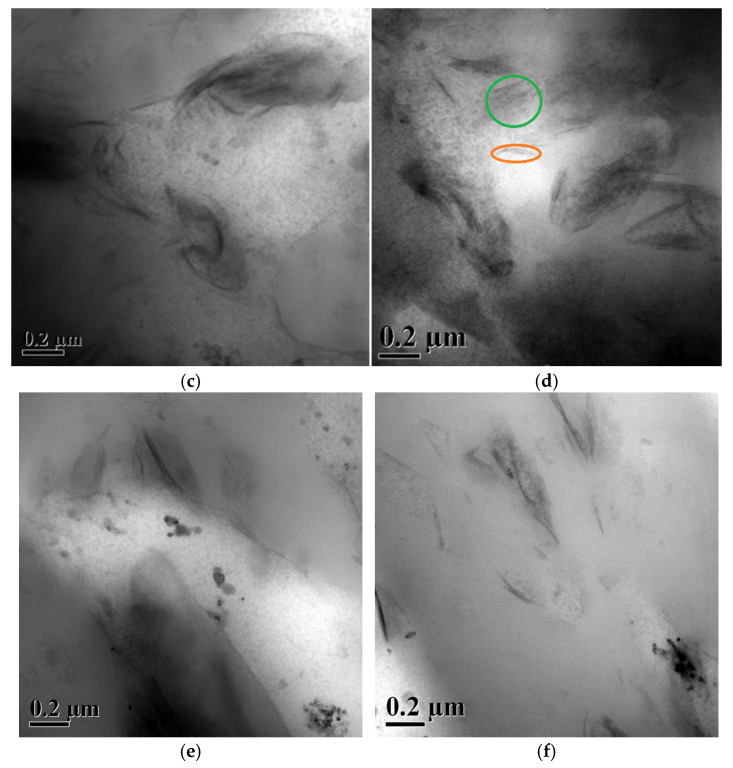
TEM images of 70/30 (10) NR/NBR blend nanocomposites (**a**,**b**); 50/50 (10) NR/NBR blend nanocomposites (**c**,**d**) with green and orange ovals highlighting intercalated and exfoliated morphology, respectively; (**e**,**f**) 30/70 (10) NR/NBR blend nanocomposites.

TEM images (Figure 13) confirm the above discussion about the intercalated/exfoliated morphology and the presence of nanoclay at the interface, supporting the conclusion that filler–polymer interfacial interactions play a critical role in heat transfer. The morphological features of the NR/NBR blend nanocomposites at a constant filler loading of 10 phr were examined to understand the role of blend composition on phase continuity, filler dispersion, and interfacial interactions. Representative micrographs for 70/30, 50/50, and 30/70 NR/NBR compositions reveal a strong dependence of microstructure on blend ratio. For the 70/30 NR/NBR blend, (Figure 13a,b) the morphology is dominated by a continuous NR-rich matrix with dispersed NBR domains. The filler particles are preferentially distributed within the NBR-rich phase, with limited penetration into the minor NR phase. This selective localization leads to localized filler-rich regions rather than a fully interconnected network, which explains the comparatively lower contribution to strain-induced reinforcement and hysteresis modulation observed in this composition at 10 phr. The 50/50 NR/NBR blend (Figure 13c,d) exhibits a co-continuous morphology, where both NR and NBR phases form interconnected networks. Notably, the filler particles are uniformly distributed along the interphase region and within both polymer phases, indicating strong filler–polymer affinity and interfacial stabilization. Evidence of filler bridging across the NR/NBR interface suggests the formation of an interface where clay nanoparticles get localized at the interphase of co-continuous network, which effectively compatibilize the two polymer phases. Although partial filler agglomeration is evident at 10 phr, leading to a reduction in effective elastic reinforcement as reflected in the storage modulus, the increased hysteresis loss arises from enhanced filler–filler friction with agglomerated structures. For the 30/70 NR/NBR blend (Figure 13e,f) the morphology changes, with NBR forming the continuous phase and NR appearing as dispersed domains. Although filler dispersion remains relatively uniform, signs of partial agglomeration and reduced interfacial anchoring are visible compared to the 50/50 system. The reduced continuity of the NR phase limits the formation of an extended filler network spanning both phases, thereby weakening the synergistic reinforcement effect seen in the co-continuous blend. The morphological observations clearly demonstrate that the 50/50 NR/NBR blend provides the most favourable microstructure, characterized by the co-continuity of phases and strong filler localization at the interface. This unique morphology facilitates the development of an effective percolated filler network; the morphology thus provides direct microstructural support for the structure–property relationships discussed earlier.

In conclusion, the reported results indicate that morphological changes in the blend play a critical role in the thermal conductivity of the obtained nanocomposite. By combining rubbers with significantly different thermal conductivities and choosing suitable amounts of nanoclay, it is possible to engineer materials with tailored heat dissipation properties, optimizing performance for diverse applications.

### 3.10. Thermal Conductivity: Effect of Clay Modification

**Figure 14:**
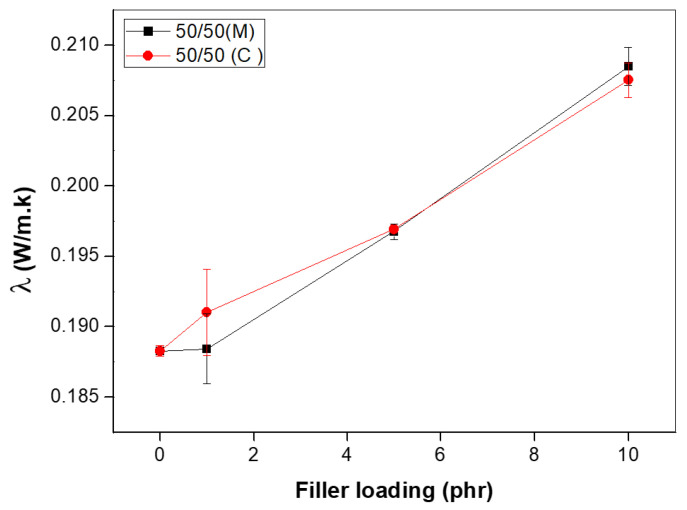
Thermal conductivity vs. filler loading for O1Mt and O2K filled NR/NBR nanocomposites.

Finally, to evaluate the possible influence of different nanoclay modifications, the thermal conductivity values of the 50/50 NR/NBR blend nanocomposite with the two different nanoclays were observed and are provided in Figure 14. It was found that the thermal conductivity is not much affected by the clay modification.

## 4. Conclusions

In this paper, a thorough investigation of the rheological, viscoelastic, and thermal properties of nanoclay-filled NR/NBR nanocomposites was performed. Different blend compositions, as different nanofiller loadings and even different organic modifications of the nanoclay, were considered.

- Rheological curves showed an increase in the complex viscosity and non-Newtonian behaviour upon increasing the nanoclay content.
- Storage modulus of the systems with stronger matrix–filler interactions substantially increased with the presence of the nanoclay, indicating an increased solid-like behaviour. The presence of the nanoclay led, in some cases, to enhancements even approximating one order of magnitude.
- Pure elastomer nanocomposites showed the Payne effect, increasing on increasing the nanofiller content.
- Damping behaviour, related to hysteresis loss, increased at higher nanoclay contents (with hysteresis loop increasing tenfold going from 50/50 blend to 50/50(10) nanocomposite) and at subsequent cycles; also, the blend composition affected the behaviour, showing higher hysteresis loss at higher NR fractions.
- Thermal properties were found to significantly change with the blend composition and the nanofiller amount; in particular, the thermal conductivity increased with the NBR content (about 15% going from 0 to 50% NBR amount), as well as with the clay amount (up to approx. 10% increase with 10% filler inclusion). This was explained by considering the different thermal conductivities of the two rubbers and the observed co-continuous morphology of the blends, with high contact area between the polymer and nanoclay improving the rate of heat transfer across the interface.
- Different clay modification could significantly affect the hysteresis loss (with loss more than doubled in O2K samples), but not the overall thermal conductivity.

The results show that nanoclay can act as an active structural element in NR/NBR blends, inducing filler–filler and filler–polymer three-dimensional networks that govern flow behaviour, elastic reinforcement, energy dissipation, and heat transfer. These findings provide support for tailoring viscoelastic damping and thermal performance of rubber nanocomposites through varying blend composition, nanofiller loading, and clay surface modification.

## Figures and Tables

**Table 1:** Characteristics of NR.

Property	Value
Mooney Viscosity	85 mL (1 + 4) at 100 °C
Weight average molar mass (Mw)	~780,000 g/mol
Number average molar mass (Mn)	~260,000 g/mol
Specific gravity (g/cm^3^)	0.98
Polydispersity Index (Mw/Mn)	~3
Dirt percent by mass (Max)	0.05%
Volatile matter, percent by mass (Max)	0.8%
Ash, percent by mass (Max)	0.6%
Nitrogen, percent by mass (Max)	>0.6%
Initial plasticity (Po)	>30 min
Plasticity retention index (PRI)	60 min

**Table 2:** Characteristics of NBR.

Property	Value
ACN	33%
Specific gravity (g/cm^3^)	0.98
Mooney Viscosity	40 mL (1 + 4) at 100 °C
Ash	0.8–1.4%
Volatiles	0.5%
Type	Cold

**Table 3:** Properties of layered silicate—Cloisite 10A.

Property	Value
Organic modifier	Dimethyl benzyl hydrogenated Tallow modified montmorillonite (HT is Tallow (~65% C18; ~30% C16; ~5% C14)
Color	Off white
Modifier concentration	125 (meq/100 g clay)
Packed bulk density	265 g/L
d-spacing (d_100_)	19.22 (Ǻ)
Moisture	<3%
Typical dry particle size	<10 μm (d_50_)
Density	1.9 g/cm^3^

**Table 4:** Curing agents, additives, and formulations.

Materials	Amount, Parts per Hundred Rubber (phr) (in the Order of Mixing)
Zinc oxide	2.5
Stearic acid	1.5
CBS (N-cyclohexyl-benzothiazyl sulphonamide)	1.3
TMTD (tetra methyl thiuram disulphide)	0.2
Sulphur	2.25

**Table 5:** Area inside the hysteresis loop for different NR/NBR nanocomposites.

Filler Loading (phr)	Area Inside the Loop (a.u.)			
	100/0	70/30	50/50	0/100
0	0.53	1.07	0.70	1.62
1	1.88	1.25	2.09	2.47
2	2.36	2.05	2.12	3.53
5	1.58	3.53	4.49	8.11
10	4.40	6.58	8.25	6.5

**Table 6:** Area inside the hysteresis loop for three consecutive loading–unloading cycles (70/30-5 NR/NBR clay nanocomposites) with two different nanoclay types (O1Mt, O2K).

Sample	Cycle 1 Area (a.u.)	Cycle 2 Area (a.u.)	Cycle3 Area (a.u.)
70/30(5) O1Mt	0.65	0.82	1.94
70/30(5) O2K	0.14	2.36	4.75

## Data Availability

Data can be made available from the corresponding authors under reasonable request. The raw data supporting the conclusions of this article will be made available by the authors on request.
